# Soil Microbial Community Structure and Metabolic Activity of *Pinus elliottii* Plantations across Different Stand Ages in a Subtropical Area

**DOI:** 10.1371/journal.pone.0135354

**Published:** 2015-08-12

**Authors:** Zeyan Wu, Stacey Elizabeth Haack, Wenxiong Lin, Bailian Li, Linkun Wu, Changxun Fang, Zhixing Zhang

**Affiliations:** 1 Fujian Agriculture and Forestry University, Fujian, China; 2 Department of Plant Pathology and Microbiology, University of California Riverside, Riverside, California, United States of America; 3 Ecological Complexity and Modeling Laboratory, University of California Riverside, Riverside, California, United States of America; Shanxi University, CHINA

## Abstract

Soil microbes play an essential role in the forest ecosystem as an active component. This study examined the hypothesis that soil microbial community structure and metabolic activity would vary with the increasing stand ages in long-term pure plantations of *Pinus elliottii*. The phospholipid fatty acids (PLFA) combined with community level physiological profiles (CLPP) method was used to assess these characteristics in the rhizospheric soils of *P*. *elliottii*. We found that the soil microbial communities were significantly different among different stand ages of *P*. *elliottii* plantations. The PLFA analysis indicated that the bacterial biomass was higher than the actinomycic and fungal biomass in all stand ages. However, the bacterial biomass decreased with the increasing stand ages, while the fungal biomass increased. The four maximum biomarker concentrations in rhizospheric soils of *P*. *elliottii* for all stand ages were 18:1ω9c, 16:1ω7c, 18:3ω6c (6,9,12) and cy19:0, representing measures of fungal and gram negative bacterial biomass. In addition, CLPP analysis revealed that the utilization rate of amino acids, polymers, phenolic acids, and carbohydrates of soil microbial community gradually decreased with increasing stand ages, though this pattern was not observed for carboxylic acids and amines. Microbial community diversity, as determined by the Simpson index, Shannon-Wiener index, Richness index and McIntosh index, significantly decreased as stand age increased. Overall, both the PLFA and CLPP illustrated that the long-term pure plantation pattern exacerbated the microecological imbalance previously described in the rhizospheric soils of *P*. *elliottii*, and markedly decreased the soil microbial community diversity and metabolic activity. Based on the correlation analysis, we concluded that the soil nutrient and C/N ratio most significantly contributed to the variation of soil microbial community structure and metabolic activity in different stand ages of *P*. *elliottii* plantations.

## Introduction

*Pinus elliottii* is a tree species native to the southeastern United States, and has been widely planted in China since 1980s due to its forage characteristics of high yield and fast growth [1]. Presently, it covers 11 provinces and has become one of the most important economic tree species in China [2]. However, dramatic yield decline and soil degradation observed in long-term pure plantations of *P*. *elliottii* have attracted an increasing level of interest from many ecologists and foresters [3]. Previous studies have shown that continuous cultivation of a single species resulted in degradation of the soil [4]. This phenomenon has been observed in many cultivated tree species, such as *Cunninghamia lanceolata*, *Larix gmelinii*, *Pinus massoniana*, *Eucalyptus spp*., and *Populus spp*. [5–9]. It has also been demonstrated that soil degradation inevitably results in the imbalance of soil microbial communities [10]. In addition, it is suggested that this imbalance further exacerbates soil degradation in long-term pure plantation cultivation [11]. Due to its long-term importance as a forestry crop in China, a number of studies have addressed above-ground characteristics of *P*. *elliottii* continuous pure plantations, including their growth regulation, biomass, and allelopathy [12–13]. However, restricted by soil ecosystem complexity and reliable experimental methods, the effects of *P*. *elliottii* long-term pure plantation patterns on the below-ground soil microbial community has not yet been reported.

Soil microbes play an active and essential role in the forest ecosystem [14]. The community structure and carbon metabolic activity of microbes have been shown as key indicators of soil quality. Since soil degradation is a common phenomenon in long-term pure plantations, understanding how soil microbial communities change with stand age helps to gain a better scientific understanding of how to best manage these man-made forests. This approach has gained momentum in the scientific community, with studies conducted on a variety of annual and a few perennial species. For example, Lu *et al*. discovered that without any soil-health management practices, long-term safflower (*Carthamus tinctorius*) plantations resulted in a shift of soil fertility from high (bacteria dominant) to low (fungi dominant) over time [15]. Li *et al*. found that the amount of fungal biomass increased markedly in Chinese fir plantations over successive rotations [16]. Furthermore, many abiotic factors like climate, pH and soil properties also contribute to explain the variation of aboveground community composition [17]. The vegetation types also profoundly affect the soil microbial communities [18, 19]. However, limited information is currently available on the research of main factor affecting the soil microbial community in *Pinus elliottii* plantations.

To describe the composition of the microbial communities in forest soils, culture-independent methods have been widely applied. Compared to other methods, phospholipids fatty acid analysis (PLFA) and community level physiological profiles (CLPP) methods are quantitative, have a relatively high throughput, and allow rapid analyses for the high number of samples needed for field-based microbial ecology investigations. In this study, we addressed the hypotheses that soil microbial community structure and metabolic activity would vary with increasing stand ages in pure plantations of *P*. *elliottii*. The goal of this study was to answer two questions: (1) how different stand ages of *P*. *elliottii* plantations affect soil microbial community structure and metabolic activity? and (2) What are the possible environmental factors leading to these differences? For these objectives, PLFA combined with CLPP methods were used to detect the variation of soil microbial community structure and metabolic activity in *P*. *elliottii* plantations. Our study will help to further understand ecological linkages between plant diversity aboveground and microbial diversity underground, and will therefore facilitate the establishment of scientific-based, effective management to achieve better ecosystem balance in man-made forests.

## Materials and Methods

### Ethics Statement

This study has been approved by the Fujian Agriculture and Forestry University, which takes care of the planning and protecting of Xiqin Forest Farm. The study did not involve any endangered or protected species. All the data in this study can be published and shared.

### 2.1 Site description

The experiment was conducted at the Xiqin Forest Farm (26°40′N, 118°10′E), Fujian Province, South China, an 11.6 km^2^ forested area in the subtropical monsoon region. This is one of the earliest *P*. *elliottii* plantations in China. The annual mean temperature is 19.4°C with an extreme high and low temperature of 39.0°C and -0.6°C respectively. Annual mean relative humidity is 80.2%, and the region has an average of 84 fog days per year. Annual precipitation reaches 1817.0 mm, most of which (57.56%) falls in summer (May through August). The soil is dominated by yellow-red soil.

### 2.2 Field sampling and soil physicochemical properties determination

Four *P*. *elliottii* plantations aged 3, 10, 18 and 25 years and established in 2011, 2004, 1996 and 1989, respectively, were selected for our study. We established three 20 m×20 m soil sampling plots for each stand age in September 2014. Sampling plots were selected to have similar environmental characteristics such as altitude, slope position, and slope aspect. Soil samples were randomly collected from 0–20 cm depths in each plot using a soil core sampler (diameter of 2.0 cm). Twenty cores were mixed into one soil sample, which was then sieved (2 mm) to remove soil impurities, hand-mixed and stored in plastic bags. Half of each soil sample was stored at 4°C for microbe characteristics analysis, and the other half was air-dried and sieved to determine soil pH, total organic carbon (TOC), total nitrogen (TN), available nitrogen (AN), total phosphorus (TP), available phosphorus (AP), total potassium (TK), and available potassium (AK) as described by Wu *et al*. [20]. The C/N ratio was also calculated.

### 2.3 Analysis of phospholipid fatty acid (PLFA)

PLFAs were extracted and derivatized as described by Zelles *et al*. [21]. Phospholipids were methylated by methanolic KOH to form fatty acid methyl esters (FAMEs), and then analyzed using a 450GC/240MS system according to the procedure described by Patra *et al*. [22]. We used 19:0 of known concentration as an internal standard to quantify other PLFAs. The detailed experimental procedure was shown in S1 Appendix. The classification standard of indicative PLFAs representing gram-positive bacteria (Gram (+)), gram-negative bacteria (Gram (-)), actinomycetes, fungi and protozoans was carried out according to McKinley *et al*., Huygens *et al*. and Brockett *et al*. [23–25]. The Gram (+)/Gram (-) PLFA ratio and fungal/bacterial PLFA ratio (F/B) were also calculated.

### 2.4 Analysis of community level physiological profiles (CLPP)

Community level physiological profiling (CLPP) owes its beginnings to the development of the BIOLOG system in the late 1980s. In this study, the microbial catabolic diversity was assessed using BIOLOG Eco Microplate system, which contains 31 different carbon substrates, allowing triplicate samples arranged on a single 96-well plate [26]. The optical density (OD) at 590 nm was recorded at 24 h-intervals by automatic microplate reader in order to calculate the average well-color development (AWCD). The average well-color development is the sum of all 31 substrates’ utilization values by culturable bacteria, divided by 31. Based on the examination of the kinetic curves of the AWCD, 96 h measurements were chosen for further data analysis. The AWCD was determined as described by Wu *et al*. [20]. The detailed experimental procedure was shown in S2 Appendix.

### 2.5 Statistical analysis

Microbial activity in each microplate, expressed as average well-color development (AWCD) was determined by the equation $AWCD=\sum \frac{C-R}{31}$, where *C* was the optical density within each well and *R* was the absorbance value of the control well. To test significant differences among soil samples, we performed one way analysis of variance (ANOVA) followed by Least Significant Difference (LSD) tests (P < 0.05) using DPS software version 7.05. Principal component analysis (PCA) and correlation analysis were performed using SPSS11.5 software.

## Results

### 3.1 Soil physicochemical properties

Most soil physicochemical properties selected for analysis in this study were significantly different among the soil samples (*P*<0.05, Table 1). The pH values ranged from 5.78 to 6.36, indicating that all test soils were acidic. A significant decrease was observed in TOC, AN, AP, and TK with increasing stand ages. These indicator properties in 3 year stand soil were 34.27%, 91.48%, 89.89%, and 71.85%, more than that in 25 year stand soil respectively. Conversely, the value of C/N ratio increased with the increasing stand ages.

**Table 1:** Soil physicochemical properties in different stand ages of *P*. *elliottii* plantations.

Stand ages	Ph	TOC/ (g·kg^-1^)	TN/ (g·kg^-1^)	AN/ (mg·kg^-1^)	TP/ (g·kg^-1^)	AP/ (mg·kg^-1^)	TK/ (mg·kg^-1^)	AK/ (mg·kg^-1^)	C/N
**3**	6.36a	21.86a	1.12a	38.22a	0.56a	3.76a	23.99a	109.11a	19.51d
**10**	6.01b	19.54b	0.95b	34.09b	0.53a	3.05b	20.06b	89.17c	20.56c
**18**	5.82c	17.37c	0.81c	27.84c	0.41b	2.67c	19.97c	96.24b	21.44b
**25**	5.78c	16.28d	0.75c	19.96d	0.33c	1.98d	13.96d	76.08d	21.70a

Note: Values within a column followed by the same letter are not significantly different at *P*<0.05. The same is true in subsequent table columns.

### 3.2 PLFA determination of microbial community composition

Our results demonstrated a significant effect of stand age on soil microbial community composition. The Gram (+) bacteria, Gram (-) bacteria, Fungi, and actinomycete components are listed in Table 2, which according to S1 Table. A total of 18 different PLFAs were identified in this study. Among them, 10Me17:0 was only found in the 3 year stand soil, while 16:1ω9c was only found in 3 and 10 year stand soil. All other PLFAs were detected in all stand age soils. The content of bacterial PLFAs was highest in all soil samples, followed by fungi, and actinomycetes were the least detected. Total PLFAs, Gram(+) bacteria and Gram(−) bacteria all decreased with increasing stand ages, with values in 3 year stand soil equaling 29.68%, 127.79% and 81.96%, higher than that in 24 year stand soil respectively. Many bacterial PLFAs showed the same trend, including 16:1ω7c, 16:00, cy19:0, i18:0, 18:00, cy17:0, i17:0 and i17:0. The Gram (+)/Gram (-) ratio was less than 1 in all soil samples, indicating that the abundance of Gram (+) PLFAs was higher than that of Gram (-) PLFAs. The amount of fungal PLFAs showed an opposite trend, increasing across stand ages, resulting in a corresponding increase in the fungi/bacteria ratio as well. Actinomycete levels did not change significantly across the stand ages.

**Table 2:** Types and contents of PLFAs in different stand ages of *P*. *elliottii* plantations (ug·g^-1^).

No.	Biomarkers	Microbial group	Stand ages			
			3	10	18	25
**1**	18:1ω9c	Fungi	6.91d	8.23c	13.78b	15.26a
**2**	16:1ω7c	Gram(-) bacteria	11.36a	9.21b	7.94c	6.44d
**3**	i14:0	Gram(+) bacteria	3.59a	2.88b	2.41c	2.38c
**4**	10Me18:0	Actinomycete	4.66a	3.54b	3.45b	3.06c
**5**	16:00	Gram(-) bacteria	7.55a	5.79b	4.32c	2.96d
**6**	cy19:0	Gram(-) bacteria	25.67a	23.93b	19.26c	15.17d
**7**	18:3ω6c(6,9,12)	Fungi	9.74d	11.43c	14.94b	15.85a
**8**	16:1ω9c	Gram(+) bacteria	4.09a	2.56b	-	-
**9**	i18:0	Gram(+) bacteria	5.66a	4.78b	4.04c	3.44d
**10**	a17:0	Gram(+) bacteria	3.31a	2.79b	2.54c	2.46c
**11**	10Me16:0	Actinomycete	3.93a	3.27c	3.44b	3.85a
**12**	18:00	Gram(-) bacteria	2.73a	2.34b	1.25c	0.76d
**13**	cy17:0	Gram(-) bacteria	8.77a	7.81c	7.96b	5.49d
**14**	18:3ω3	Fungi	1.67d	1.94c	2.53b	3.52a
**15**	10Me17:0	Actinomycete	1.42	-	-	-
**16**	i17:0	Gram(+) bacteria	1.93a	1.78b	1.59c	1.16d
**17**	20:4ω6c(6,9,12,15)	Protozoon	0.58b	0.74a	0.51b	0.83a
**18**	15:0	Gram(+) bacteria	4.45a	3.78b	0.93c	0.67d
**19**	-	Total PLFA	108.02a	96.80b	90.89c	83.30d
**20**	-	Bacteria	79.11a	67.65b	52.24c	40.93d
**21**	-	Gram(+)	23.03a	18.57b	11.51c	10.11d
**22**	-	Gram(-)	56.08a	49.08b	40.73c	30.82d
**23**	-	Fungi	18.32d	21.60c	31.25b	34.63a
**24**	-	Actinomycete	10.01a	6.81b	6.89b	6.91b
**25**	-	Gram (+) / Gram (-) (%)	41.06a	37.84b	28.26d	32.80c
**26**	-	Fungi/ Bacteria (%) (F/B)	23.16d	31.93c	59.82b	84.61a

**Fig 1:**
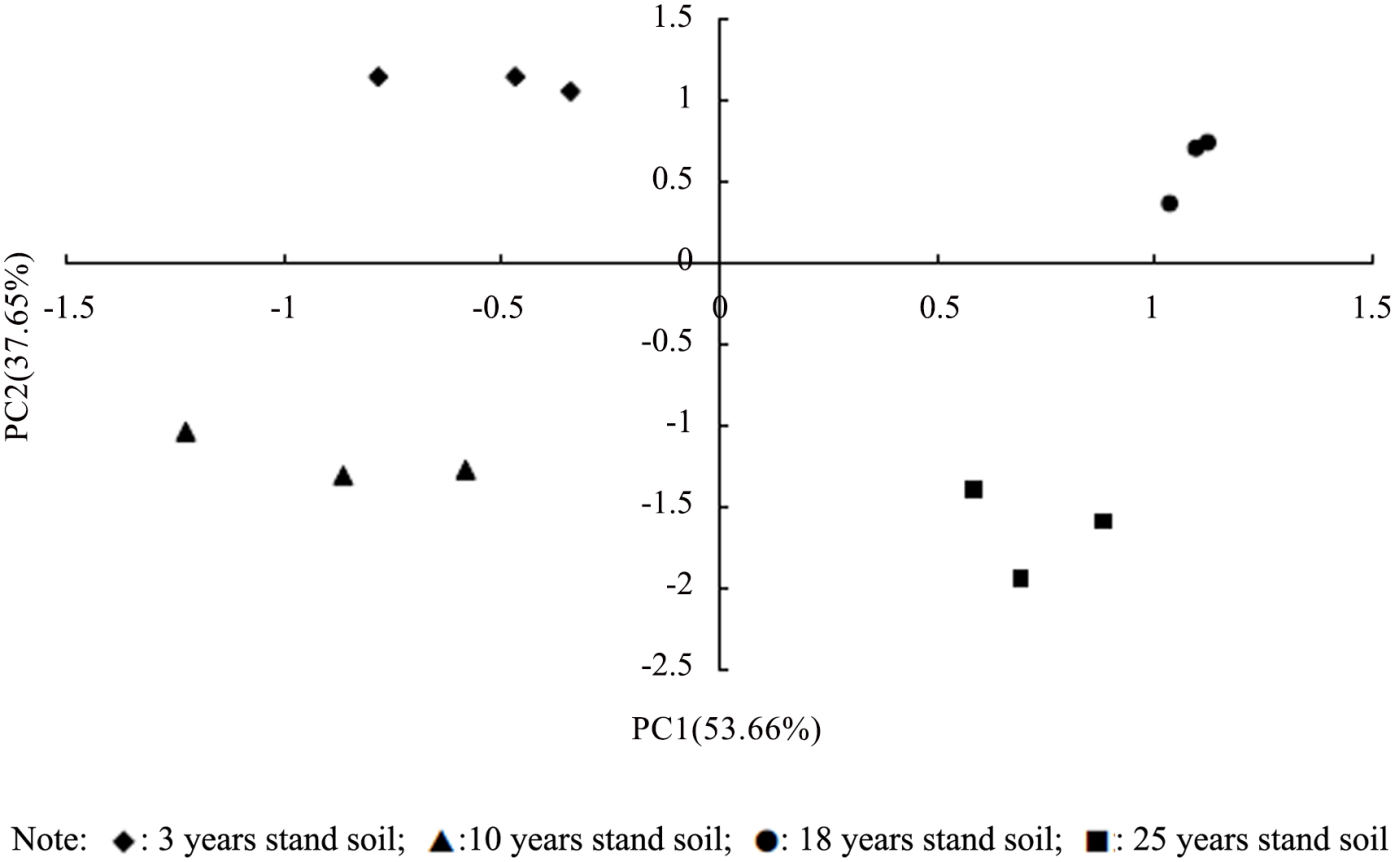
Principal component analysis on PLFAs of different microbial groups in soils. The figure describes the variance of soil microbial communities in the different stand ages of *P*. *elliottii* were clearly different from each other.

The content of all individual PLFAs was subjected to the principal component analysis (PCA) (Fig 1). Four soil samples were clearly distinguishable along the first principal component of the score plot, with 3 year stand soil and 10 year stand soil on the left side and 18 year stand soil and 25 year stand soil on the right side of the axis. PC1 accounted for 53.66% and PC2 accounted for 37.65% of the total variation, For PC1, lipid signatures cy19:0, a17:0, 18:1ω9c and 16:1ω7c had the highest loading scores, whereas for PC2, i14:0, i18:0 and cy17:0 had the highest loading scores.

### 3.3 Analysis of community level physiological profiles (CLPP)

**Fig 2:**
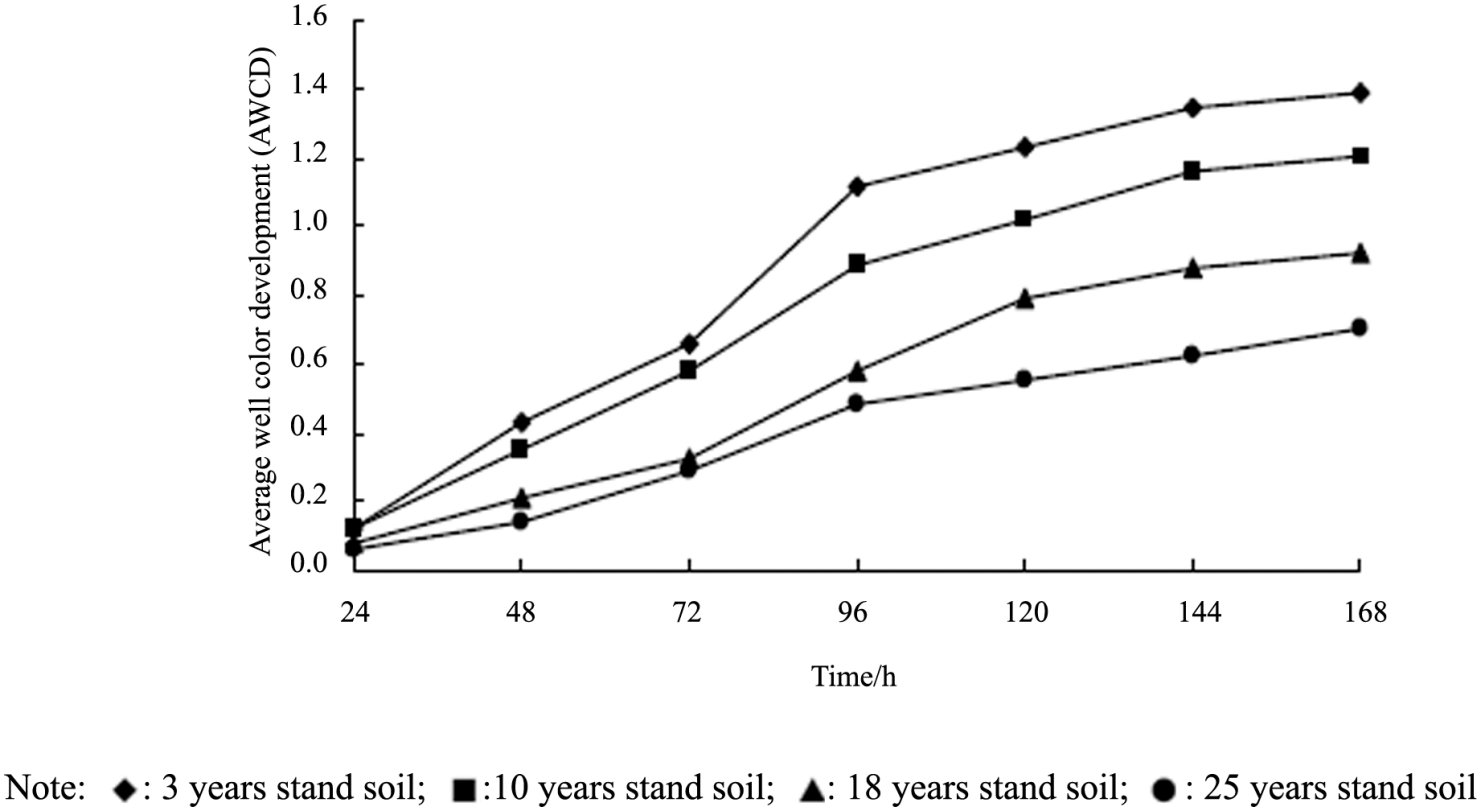
Changes of AWCD of soil microbe community with incubation time in soil samples of different stand ages. The figure describes the AWCD of different stand soil change with incubation time, which were clearly different from each other.

The AWCD values, as a measure of the total microbial activity, increased and showed a typical sigmoid course curve across the 168 h incubation for all samples (Fig 2, S2 Table). However, significant differences were observed in microbial activity rate of increase among the samples. The 3 year stand soil showed the highest utilization rate as indicated by a steep slope, whereas the 25 year stand soil showed the lowest utilization rate as indicated by a more gradual slope. Overall, the AWCD values significantly decreased with each increase in stand age measured (3 years > 10 years > 18 years > 25 years).

**Fig 3:**
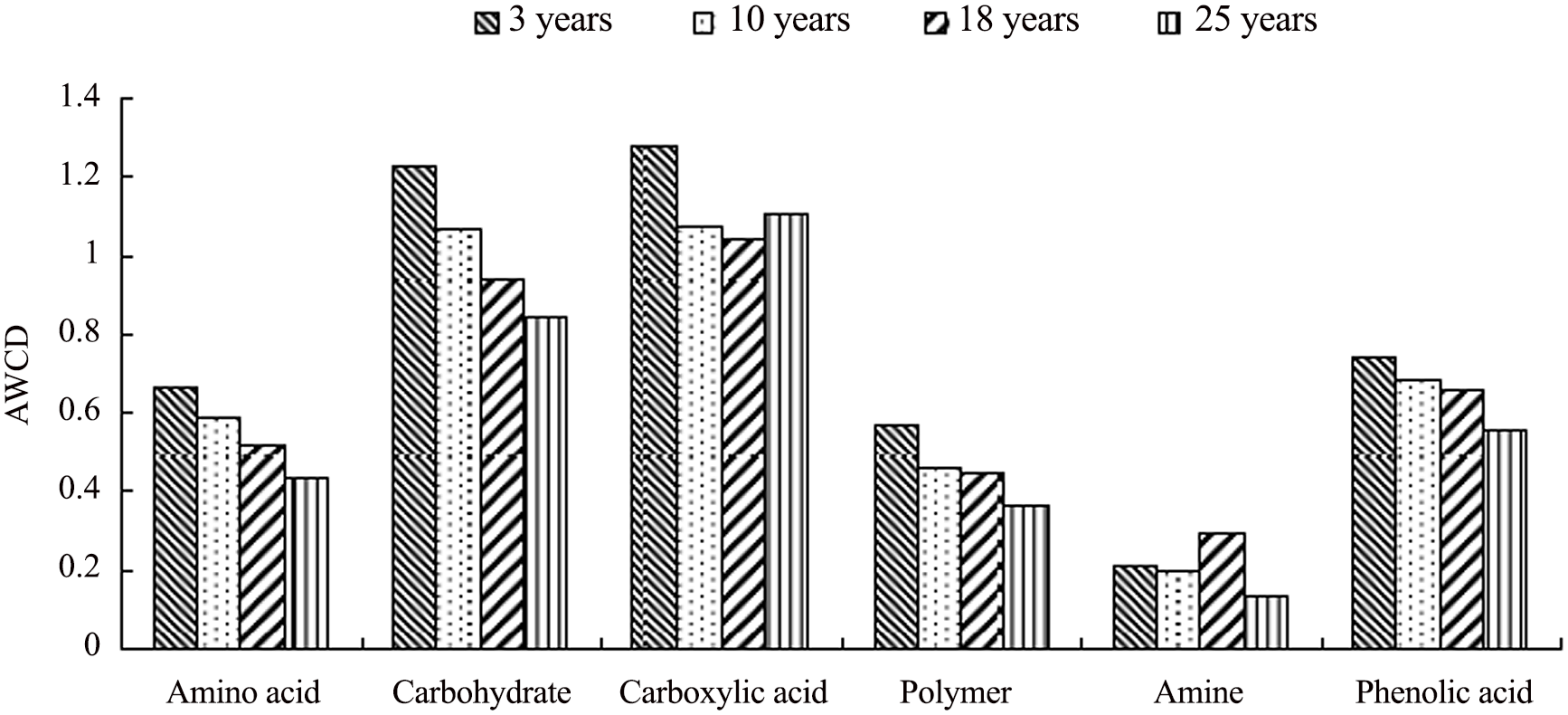
Carbon source utilization by soil microbial community at different stand ages of *P*. *elliottii* plantations. The figure describes the carbon source utilization of different stand soil microbial communities were clearly different from each other.

We divided 31 single carbon substrates into 6 categories: amino acids, polymers, phenolic acids, amines, carboxylic acids and carbohydrates as described by Chowdhury and Dick [27]. Overall, the sum of AWCD values within all 6 categories significantly decreased with each increase in stand age (3 years > 10 years > 18 years > 25 years) (Fig 3). Except for carboxylic acids and amines, the 96 h AWCD values of amino acid, carbohydrate, polymer and phenolic acid carbon substrate groups were highest in the 3 year stand soil and lowest in the 25 year stand soil. The levels of amino acids, carbohydrates, polymers, amines and phenolic acids of microbial communities from the 10 year and 18 year stand soil were higher than those from the 25 stand year soil. The Simpson index, Shannon-Wiener index, Richness index and McIntosh index in 3 year stand soil were all higher than that in 25 year stand soil, and these values also followed a significant sequence order 3 years > 10 years > 18 years > 25 years (Table 3).

**Table 3:** Functional diversity indexes of soil microbial communities at different stand ages.

Stand age	Simpson index	Shannon index	Richness index	McIntosh index
**3 years**	0.974	3.449	17.33	0.933
**10 years**	0.926	3.286	15.19	0.876
**18 years**	0.908	3.193	13.72	0.768
**25 years**	0.875	3.051	11.09	0.669

### 3.4 The relationship between microbial community diversity and soil properties

Table 4 shows the relationship between microbial community diversity and soil properties. TOC and AN showed a significant positive correlation with the microbial community diversity, while C/N ratio showed a significant negative correlation. This indicates that soil C and N contents are very important factors to soil microbial diversity.

**Table 4:** Correlation analysis of microbial community diversity and soil properties.

	Simpson index	Shannon index	Richness index	McIntosh index
**pH**	-0.395	-0.347	-0.384	-0.290
**TOC/ (g·kg** ^**-1**^ **)**	0.956**	0.981**	0.947**	0.965**
**TN/ (g·kg** ^**-1**^ **)**	0.679	0.572	0.625	0.448
**AN/ (mg·kg** ^**-1**^ **)**	0.873*	0.921**	0.851*	0.890*
**TP/ (g·kg** ^**-1**^ **)**	0.709	0.878*	0.842*	0.731
**AP/ (mg·kg** ^**-1**^ **)**	0.930**	0.755	0.801	0.819
**TK/ (mg·kg** ^**-1**^ **)**	0.871*	0.830	0.744	0.731
**AK/ (mg·kg** ^**-1**^ **)**	0.677	0.938**	0.885*	0.711
**C/N**	-0.926**	-0.907**	-0.949**	-0.980**

Note:

*means *P* < 0.05, significant correlation;

**means *P* < 0.01, extremely significant correlation.

## Discussion

Our study demonstrated that soil microbial community diversity and metabolic activity decreased with increasing stand ages in *P*. *elliottii* plantations. The bacterial biomass decreased with the increasing stand ages, while the fungal biomass increased. The carbon source utilization rate and community diversity of soil microbes gradually decreased with increasing stand ages. Overall, both the PLFA and CLPP illustrated that the long-term pure plantation pattern exacerbated the microecological imbalance previously described in the rhizospheric soils of *P*. *elliottii*, and markedly decreased the soil microbial community diversity and metabolic activity. Similar trends were also reported in other tree species [28]. The importance of soil nutrient content in shaping microbial communities has been reported by a number of studies, and has been established as a key determinant for soil microorganism survival, species composition, and metabolism [6]. Therefore, the most important contributor to our observed reduction in microbial diversity and metabolic activity in *P*. *elliottii* stands is the decline of soil nutrient content. *P*. *elliottii* is a fast-growing tree species, and has a high requirement and utilization rate of soil nutrients to maintain this growth. Although litterfall can return nutrients to the soil and improve fertility to some extent, this rate of natural nutrient cycling is too slow [1]. In this study, the analysis of soil physicochemical properties showed that soil nutrient indicators such as TOC, AN, AP, and TK decreased with the increasing stand ages (Table 1). Correlation analysis also demonstrated close links between soil nutrient indicators and soil microbial diversity (Table 4). The correlation coefficient of community diversity with TOC and AN was greater than 0.945 (*p*<0.05) and 0.851 (*p*<0.05), respectively. Consequently, the decline of soil nutrient content inevitably leads to a decrease in soil microbial diversity. Furthermore, the effects of spatial change on soil nutrient content are also very important [29–32]. We will do further studies to compare the variation among different forest ecosystems in subtropical area.

In addition to nutrient content, soil C/N ratio is another important factor affecting soil microbial community structure. This study showed that the soil microbial community diversity was significantly negatively correlated with C/N ratio, indicating that high C/N ratio is not conducive to microbial growth. This result is also consistent with previous studies. For example, Högberg *et al*. found that soil C/N ratio and soil pH are important factors influencing soil microbial community composition [32]. Ushio *et al*. concluded that the variation of C/N ratio caused by tree species changed the soil microbial community structure profoundly [33]. *P*. *elliottii* litterfall contains many slowly-degrading components such as wax, cellulose, and lignin, and the C/N ratio in these substances are often relatively high [34]. Therefore, with the increasing stand ages, the accumulating litterfall increases the value of C/N ratio. Mudge *et al*. concluded the higher microbial C/N ratio indicated soil microorganisms activated by C-rich rhizodeposits with a high N demand and a greater potential for immobilization of N in the microbial biomass [35]. High C/N ratio leads to a lack of nitrogen in the soil, thereby inhibiting the activity of soil microorganisms. Furthermore, the soil tiny animals, plant root and seasonal variation might also cause or contribute to the differences. The F/B is also an indication of microbial community structure, and this proportion is known to change with changing soil environment conditions. Generally, the most abundant type of microorganism in healthy soils is bacteria, followed by actinomycetes and lastly fungi [36]. In this study, we found that during early developmental stages of *P*. *elliottii* plantations, adequate soil nutrition was beneficial to the reproduction of bacteria, which are favored in high-nutrient soil. Soil nutrient content decrease in increasing stand ages, however, resulted in an increase in fungal abundance, as fungal growth is favored in soil with lower fertility.

Our conclusions indicate that long-term monoculture plantations of *P*. *elliottii* result in a change in soil properties over time, and therefore cause a corresponding change in soil microbial community composition and metabolic activity. However, the CLPP and PLFA methods used here have limitations in their detection of soil microbial community characteristics. The experimental result of CLPP and PLFA are influenced by environmental change and physiological status of microbes under some environmental stresses. These results can be strengthened with future work combing with other soil microbial research methods such as terminal restriction fragment length polymorphism (T-RFLP) and denaturing gradient gel electrophoresis (DGGE). Our studies help to more deeply understand the ecological linkages between aboveground and belowground biota, and thereby facilitate to establishment of an effective forest management practice for the improvement of forest ecosystem balance. Based on the results as mentioned above, improved management practices, such as microbial fertilizer application, organic matter amendment and enhanced plant diversity, could be used to relieve the consecutive monoculture problems of *P*. *elliottii* in future.

## Supporting Information

S1 Appendix Phospholipid fatty acid (PLFA) analysis method. http://dx.doi.org/10.6084/m9.figshare.1423487.
(DOC)

S2 Appendix Community level physiological profiles (CLPP) analysis method. http://dx.doi.org/10.6084/m9.figshare.1423488.
(DOC)

S1 Table PLFA determination of microbial community composition. http://dx.doi.org/10.6084/m9.figshare.1423489.
(XLS)

S2 Table The optical density (OD) at 590 nm change with incubation time. http://dx.doi.org/10.6084/m9.figshare.1423490.
(XLS)
