# Supplementary material for: Soil Microbial Community Structure and Metabolic Activity of Pinus elliottii Plantations across Different Stand Ages in a Subtropical Area
Source: PLoS One. 2015 Aug 12;10(8):e0135354. doi: 10.1371/journal.pone.0135354 (PMC4533972; doi:10.1371/journal.pone.0135354)
Supplement: S1 Appendix — http://dx.doi.org/10.6084/m9.figshare.1423487. (DOC) [file pone.0135354.s001.doc]

**Phospholipid fatty acid (PLFA) analysis method**

3.0 g soil (freeze-dried) was placed in 15.8 ml mixture of citrate buffer solution, methanol, and chloroform (0.8:2.0:1.0 v/v). The sample was extracted ultrasonically and separated by centrifugation. The supernatant was transferred to a tube. The remainder was blended with 6.8 ml solution mentioned above, repeated the process. Subsequently, 4.8 ml of citrate buffer solution and 6 ml of chloroform were then added to the combined supernatant and the mixture was left to separate overnight. The chloroform phase was reduced by rotary evaporation. Phospholipids were separated from crude lipids by silica gel columns with methanol eluting.

Phospholipids were methylated by mild alkaline methanolysis (using methanolic KOH) to form fatty acid methyl esters (FAMEs). Then, FAMEs were analyzed using a 450GC/240MS system (Varian, Inc., USA) equipped with a capillary column CP8944 (30 m, 0.25 mm i.d., 0.25 m film thickness; Varian, Inc., USA). The column temperature was programmed to start at 70 ^o^C for 1 min, then ramp up at a rate of 20 ^o^C min^−1^ to 170 ^o^C which was held for 2 min, and followed by a ramp of 5 ^o^C min^−1^ to 280 ^o^C which was held for 5 min. Finally, the oven temperature was increased to 300 ^o^C at 40 ^o^C min^−1^ and held for 1.5 min. The peaks were identified based on relative retention times vs. several external standards: a mixture of 37-Component FAME Mix (47885-U, Supelco Inc., USA), a mixture of 26 Bacterial Acid Methyl Esters (47080-U, Supelco Inc., USA) and several individual FAMEs (Larodan Inc., Sweden). Individual fatty acids were quantified by comparing peak areas from the sample with peak areas of the internal standard 19:0 (nonadeconoic methyl ester) of known concentration.
