# Supplementary material for: Soil Microbial Community Structure and Metabolic Activity of Pinus elliottii Plantations across Different Stand Ages in a Subtropical Area
Source: PLoS One. 2015 Aug 12;10(8):e0135354. doi: 10.1371/journal.pone.0135354 (PMC4533972; doi:10.1371/journal.pone.0135354)
Supplement: S2 Appendix — http://dx.doi.org/10.6084/m9.figshare.1423488. (DOC) [file pone.0135354.s002.doc]

**Community level physiological proﬁles (CLPP) analysis method**

Community level physiological profiles (CLPP) were assessed by the Biolog Eco MicroplateTM system (Biolog Inc., CA, USA). Each 96 well plate consists of three replicates of 31 sole carbon substrates and 1 water blank. The plates were incubated at 25 ^o^C for 168 h, and the color development in each well was recorded as optical density (OD) at 590 nm with a plate reader (Thermo Scientific Multiskan MK3, Shanghai, China) at regular 24 h intervals. Microbial activity in each microplate, expressed as average well color development (AWCD) was determined as follows:

$$AWCD=\sum\frac{C-R}{31}$$

where *C* is the optical density within each well and *R* is the absorbance value of the control well. The 31 carbon substrates in ECO microplates were subdivided into six categories: polymers, carbohydrates, carboxylic acids, amino acids, amines and phenolic compounds.
